# Supplementary material for: Kinetic drop friction
Source: Nat Commun. 2023 Jul 29;14:4571. doi: 10.1038/s41467-023-40289-8 (PMC10387105; doi:10.1038/s41467-023-40289-8)
Supplement: Supplementary file 1 — Supplementary Information [file 41467_2023_40289_MOESM1_ESM.pdf]

Supplementary Information for

Kinetic Drop Friction

Xiaomei Li[1], Francisco Bodziony[2], Mariana Yin[2], Holger Marschall[2], Rüdiger Berger[1], Hans-Jürgen Butt[1] *

[1] Max Planck Institute for Polymer Research, Ackermannweg 10, 55128 Mainz, Germany

[2] Computational Multiphase Flows, Technische Universität Darmstadt, Alarich-Weiss-Straße 10, 64287 Darmstadt, Germany

*Corresponding author: Hans-Jürgen Butt (butt@mpip-mainz.mpg.de)

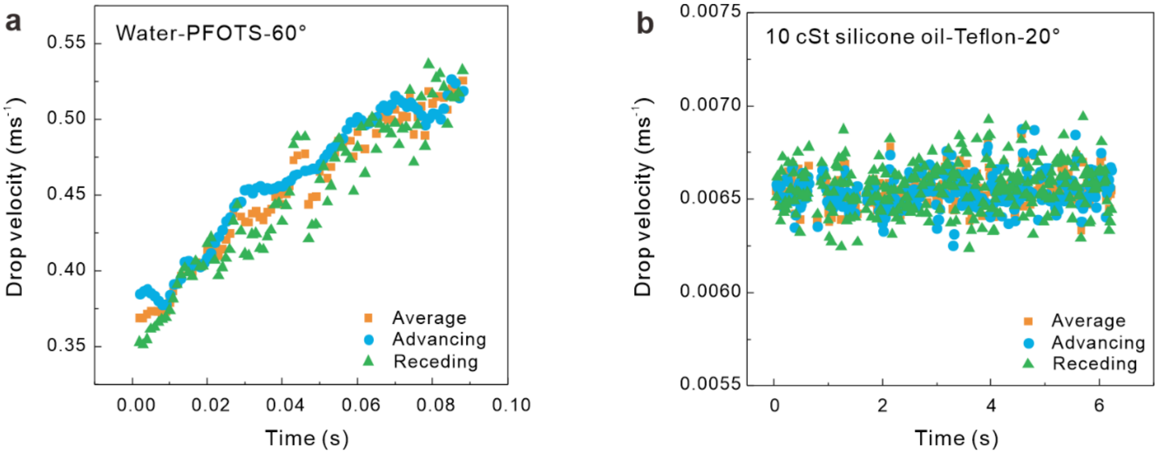

Supplementary Figure 1. Average, advancing, and receding contact-line velocity versus time for a water drop on the 60° tilted PFOTS-Si surface (a) and a 10 cSt silicone oil drop on the 20° tilted Teflon-gold surface (b). For example, the average deviation of U_{a} and U_{r} from U for a water drop sliding down the PFOTS-Si surface at 60° and for a 10 cSt silicone oil drop sliding down the Teflon-gold surfaces at 20° are (4$\pm$3) % and (3$\pm$5)%, respectively.

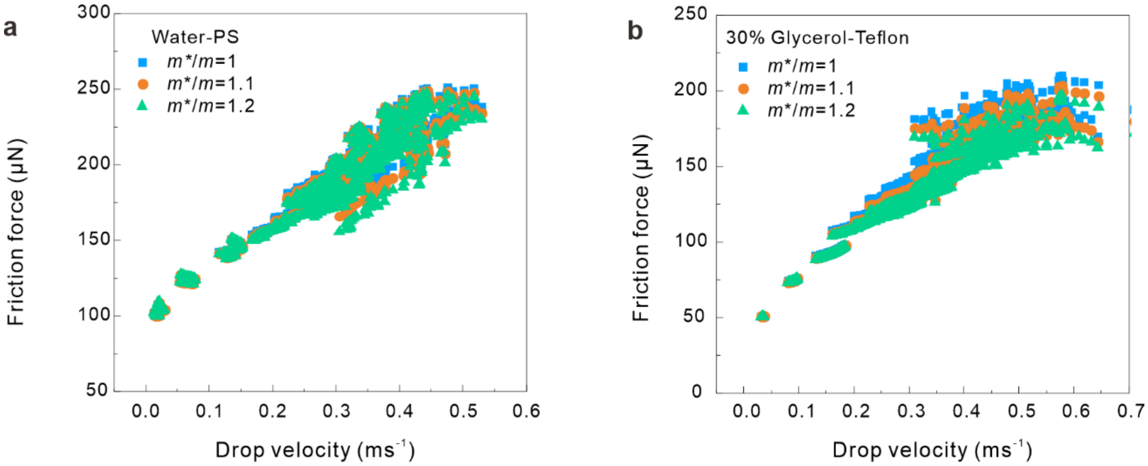

Supplementary Figure 2. Deviation of friction force when the ratio of effective mass to drop mass, m^*/m changes from 1 to 1.2. The friction forces deviate within 2.3% for the water drop on the PS-gold surface (a) and within 5% for the 30% glycerol-water mixture on the Teflon-gold surface (b) when m^*/m varies from 1.0 to 1.2. The deviation is calculated by $\frac{2(F_{\mathrm{f}}-F_{\mathrm{f}}')}{(F_{\mathrm{f}}+F_{\mathrm{f}}')}\times 100\%$ (F_{f} and F_{f}' are the force when $\frac{m^*}{m}=1$ and $\frac{m^*}{m}\neq 1$, correspondingly).

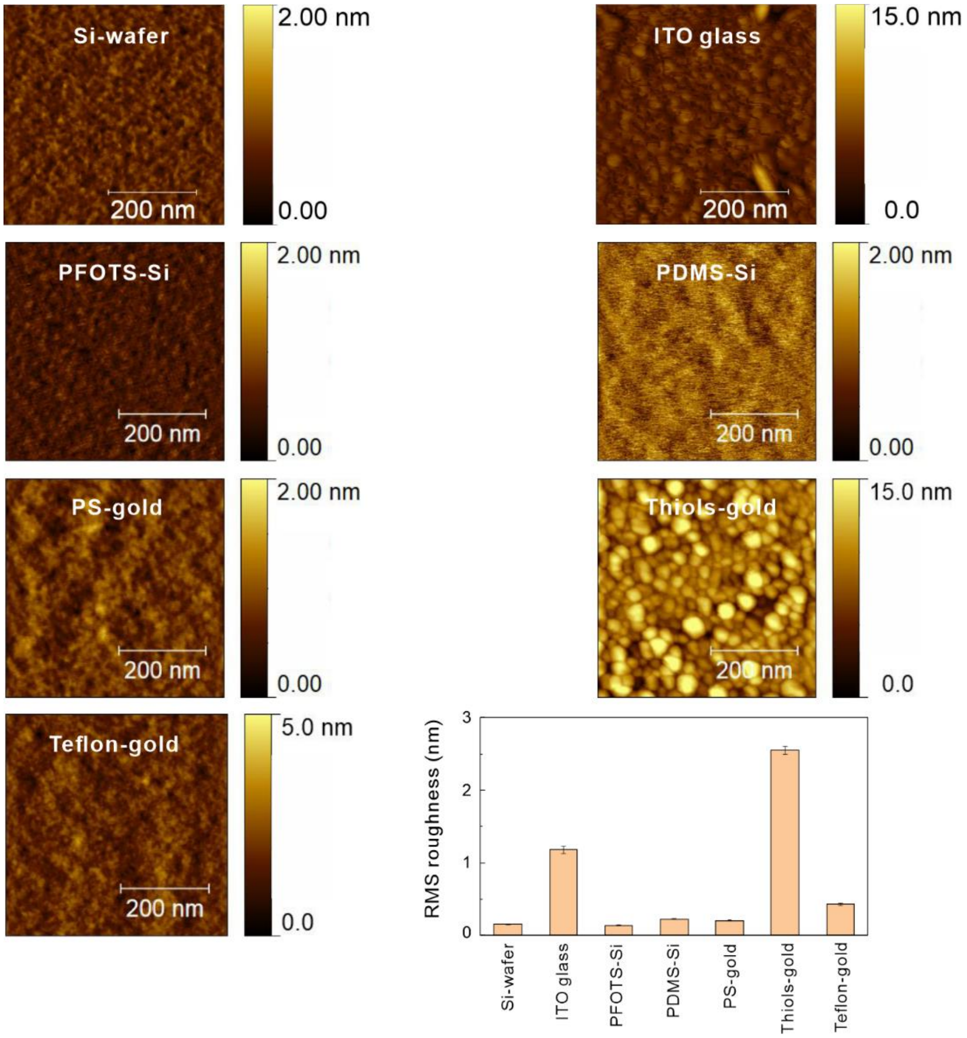

Supplementary Figure 3. Topography and average root-mean-square (RMS) roughness of all the surfaces measured by scanning force microscopy for an area of 0.5 x 0.5 μm^2. Error bars indicate the standard deviation of three measurements at different positions on the sample.

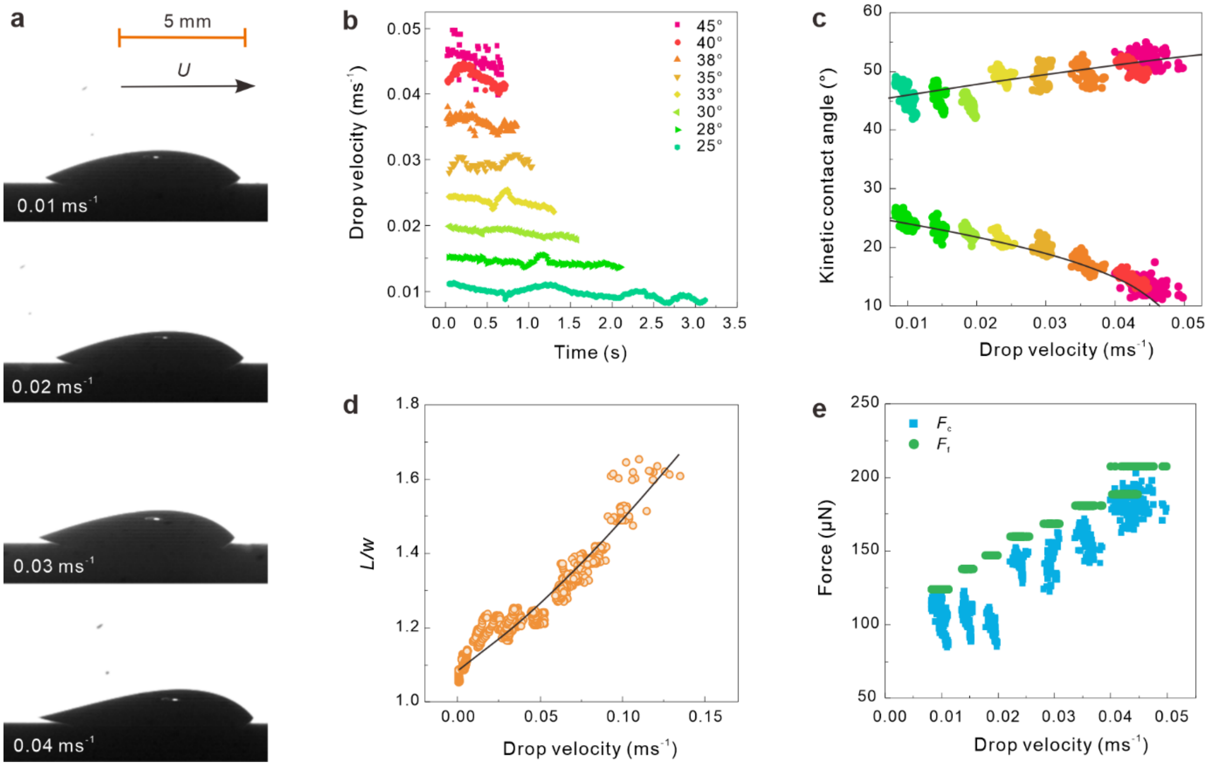

Supplementary Figure 4. Water drops on a Si wafer. (a) Drop profile at different velocities; (b) velocity of drops at different tilt angles; (c) velocity-dependent kinetic contact angle (upper data point corresponds to the advancing side, below data point corresponds to the receding side). The black curves are fitted by $\theta_{a/r} = \left(\theta_{a0/r0}^3 \pm 9\frac{U\eta}{\gamma}\ln\frac{l}{l_m}\right)^{1/3}$. The fitting parameters ($\theta_{a0}, \theta_{r0}, \frac{l}{l_m}$) are summarized in Supplementary Table 1; (d) velocity-dependent aspect ratio (length-to-width: L/w). The data was fitted with a polynom resulting in: $\frac{L}{w} = 8.312U^2 + 3.21U + 1.086$, U in ms^{-1}; (e) velocity-dependent friction force and capillary force.

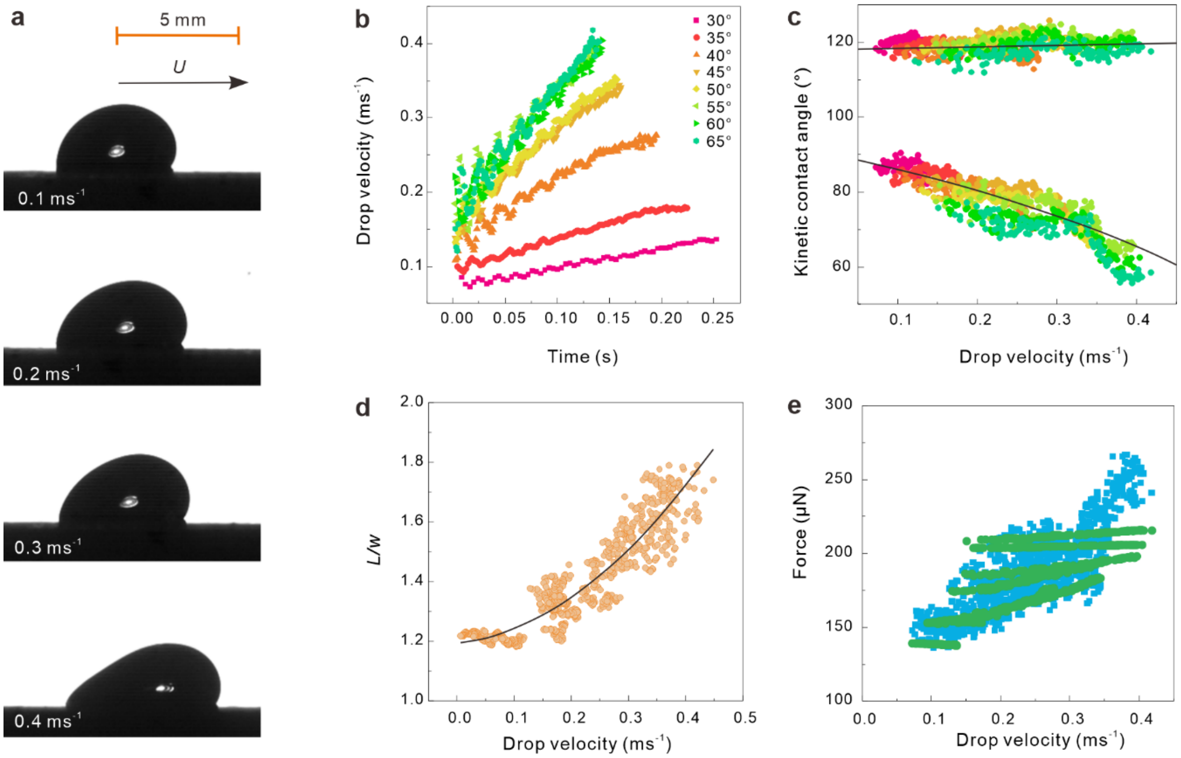

Supplementary Figure 5. Water drops on ITO glass. (a) Drop profile at different velocities; (b) velocity of drops at different tilt angles; (c) velocity-dependent kinetic contact angle (upper data point corresponds to the advancing side, below data point corresponds to the receding side). The black curves are fitted by $\theta_{\mathrm{a/r}} = \left(\theta^3_{\mathrm{a0/r0}} \pm 9\frac{U\eta}{\gamma}\ln\frac{l}{l_{\mathrm{m}}}\right)^{1/3}$. The fitting parameters ($\theta_{\mathrm{a0}}, \theta_{\mathrm{r0}}, \frac{l}{l_{\mathrm{m}}}$) are summarized in Supplementary Table 1; (d) velocity-dependent aspect ratio (length-to-width: L/w). The data was fitted with a polynom resulting in: $\frac{L}{w} = 2.782U^2 + 0.205U + 1.194$, U in $\mathrm{ms^{-1}}$; (e) velocity-dependent friction force and capillary force.

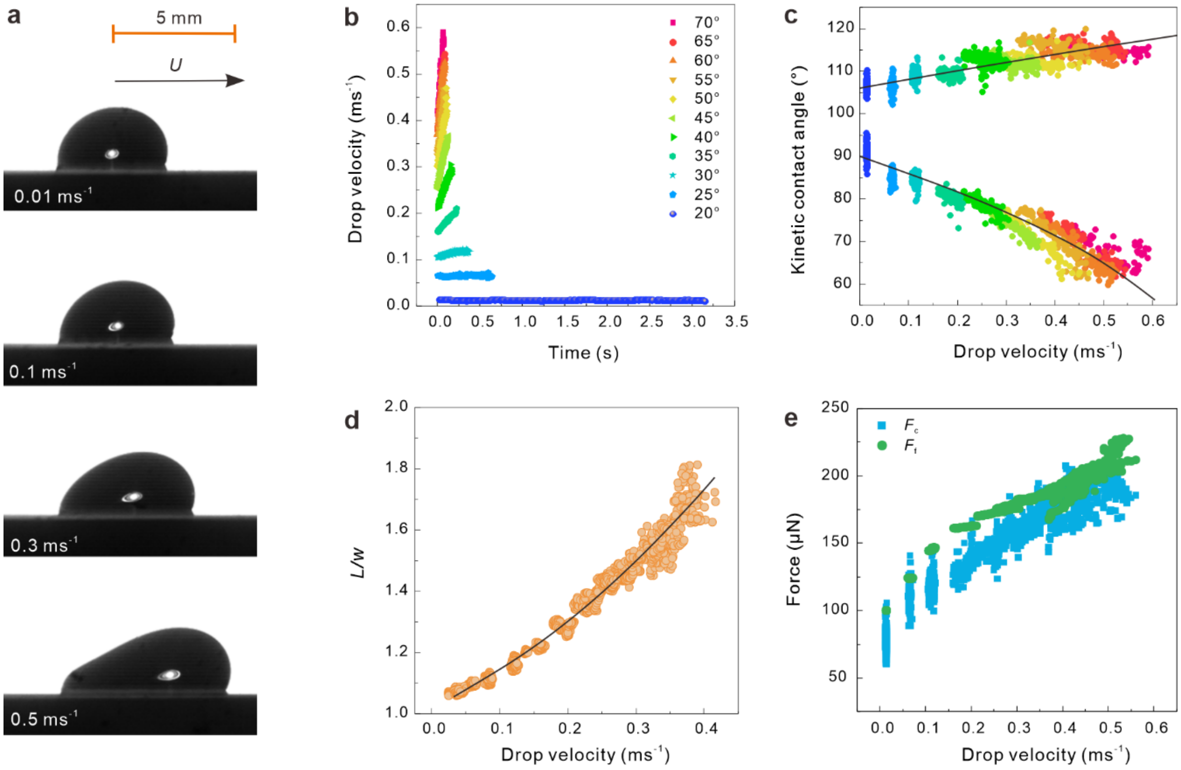

Supplementary Figure 6. Water drops on PFOTS-Si surfaces. (a) Drop profile at different velocities; (b) velocity of drops at different tilt angles; (c) velocity-dependent kinetic contact angle (upper data point corresponds to the advancing side, below data point corresponds to the receding side). The black curves are fitted by $\theta_{\mathrm{a/r}} = \left(\theta^3_{\mathrm{a0/r0}} \pm 9\frac{U\eta}{\gamma}\ln\frac{l}{l_{\mathrm{m}}}\right)^{1/3}$. The fitting parameters $(\theta_{\mathrm{a0}}, \theta_{\mathrm{r0}}, \frac{l}{l_{\mathrm{m}}})$ are summarized in Supplementary Table 1; (d) velocity-dependent aspect ratio (length-to-width: L/w). The data was fitted with a polynom resulting in: $\frac{L}{w} = 1.931U^2 + 0.993U + 1.026$, U in ms^{-1}; (e) velocity-dependent friction force and capillary force.

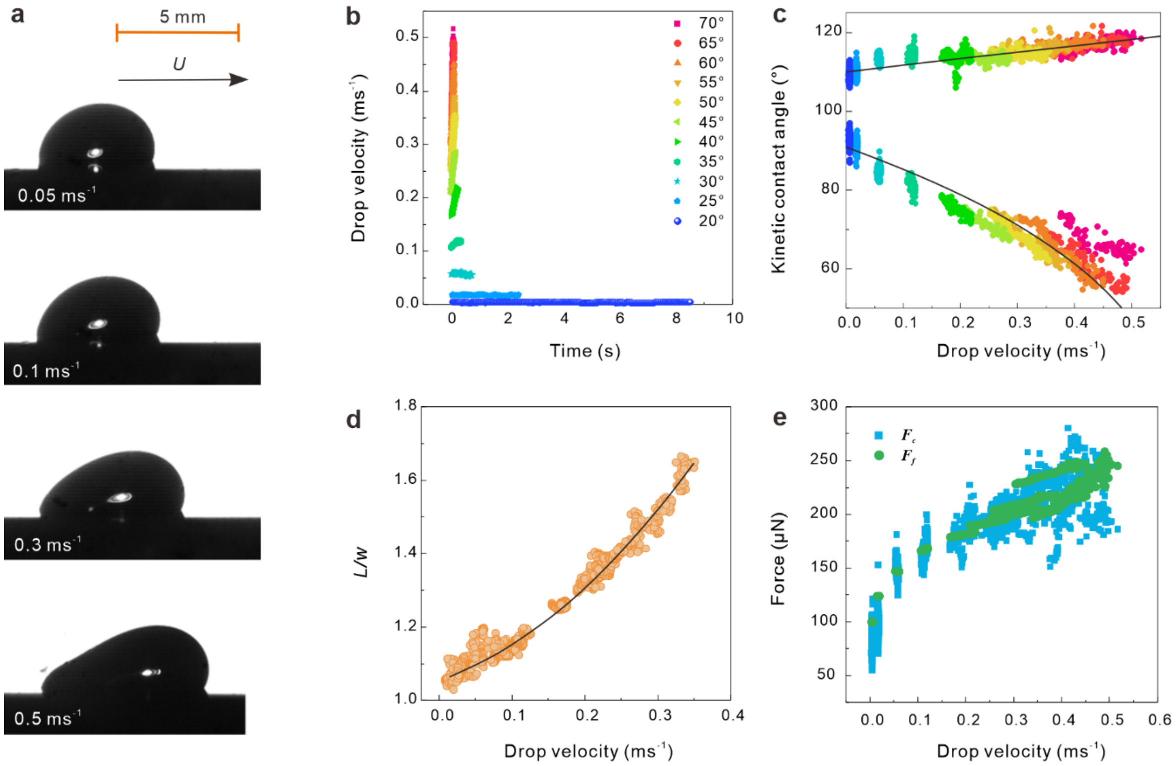

Supplementary Figure 7. Water drops on PDMS-Si surfaces. (a) Drop profile at different velocities; (b) velocity of drops at different tilt angles; (c) velocity-dependent kinetic contact angle (upper data point corresponds to the advancing side, below data point corresponds to the receding side). The black curves are fitted by $\theta_{\mathrm{a/r}} = \left(\theta^3_{\mathrm{a0/r0}} \pm 9\frac{U\eta}{\gamma}\ln\frac{l}{l_{\mathrm{m}}}\right)^{1/3}$. The fitting parameters ($\theta_{\mathrm{a0}}, \theta_{\mathrm{r0}}, \frac{l}{l_{\mathrm{m}}}$) are summarized in Supplementary Table 1; (d) velocity-dependent aspect ratio (length-to-width: L/w). The data was fitted with a polynom resulting in: $\frac{L}{w} = 2.951U^2 + 0.697U + 1.054$, U in ms^{-1}; (e) velocity-dependent friction force and capillary force.

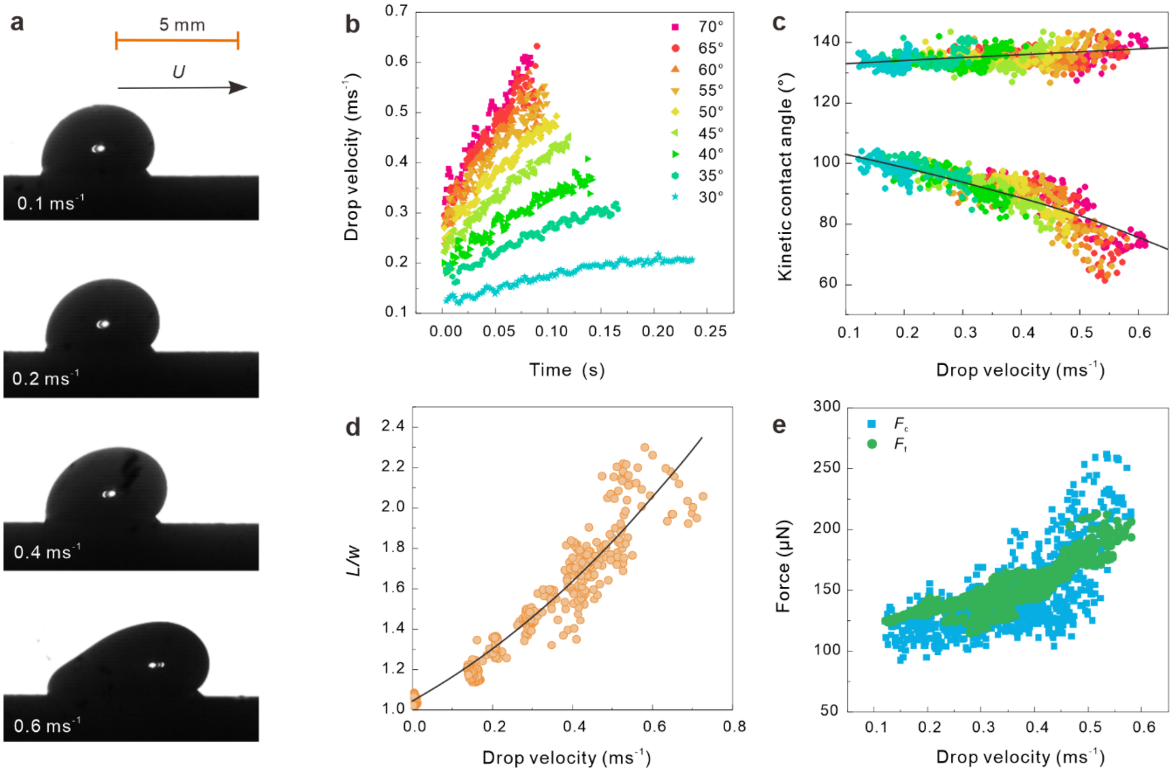

Supplementary Figure 8. Water drops on thiols-gold surfaces. (a) Drop profile at different velocities; (b) velocity of drops at different tilt angles; (c) velocity-dependent kinetic contact angle (upper data point corresponds to the advancing side, below data point corresponds to the receding side). The black curves are fitted by $\theta_{\mathrm{a/r}} = \left(\theta_{\mathrm{a0/r0}}^3 \pm 9\frac{U\eta}{\gamma}\ln\frac{l}{l_{\mathrm{m}}}\right)^{1/3}$. The fitting parameters ($\theta_{\mathrm{a0}}, \theta_{\mathrm{r0}}, \frac{l}{l_{\mathrm{m}}}$) are summarized in Supplementary Table 1; (d) velocity-dependent aspect ratio (length-to-width: L/w). The data was fitted with a polynom resulting in: $\frac{L}{w} = 1.013U^2 + 1.058U + 1.048$, U in ms^{-1}; (e) velocity-dependent friction force and capillary force.

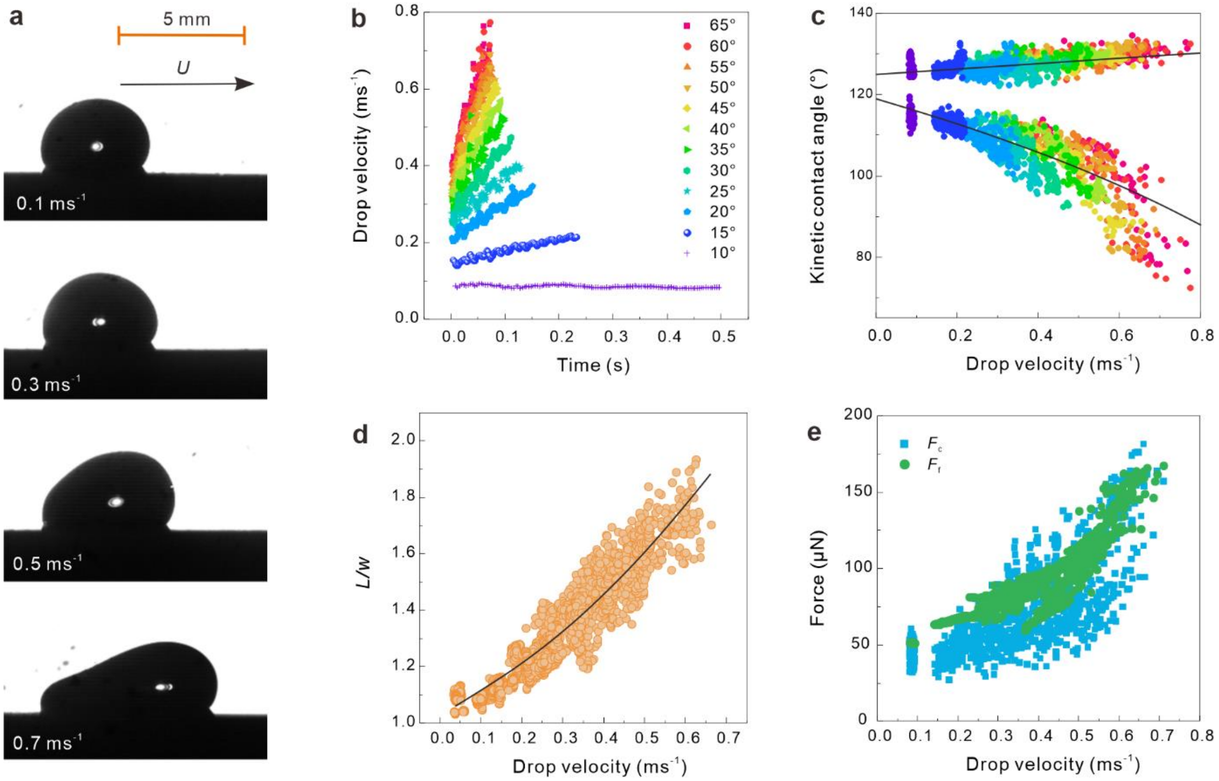

Supplementary Figure 9. Water drops on Teflon-gold surfaces. (a) Drop profile at different velocities; (b) velocity of drops at different tilt angles; (c) velocity-dependent kinetic contact angle (upper data point corresponds to the advancing side, below data point corresponds to the receding side). The black curves are fitted by $\theta_{\mathrm{a/r}} = \left(\theta_{\mathrm{a0/r0}}^3 \pm 9\frac{U\eta}{\gamma}\ln\frac{l}{l_{\mathrm{m}}}\right)^{1/3}$. The fitting parameters ($\theta_{\mathrm{a0}}, \theta_{\mathrm{r0}}, \frac{l}{l_{\mathrm{m}}}$) are summarized in Supplementary Table 1; (d) velocity-dependent aspect ratio (length-to-width: L/w). The data was fitted with a polynom resulting in: $\frac{L}{w} = 0.856U^2 + 0.715U + 1.034$, U in ms^{-1}; (e) velocity-dependent friction force and capillary force.

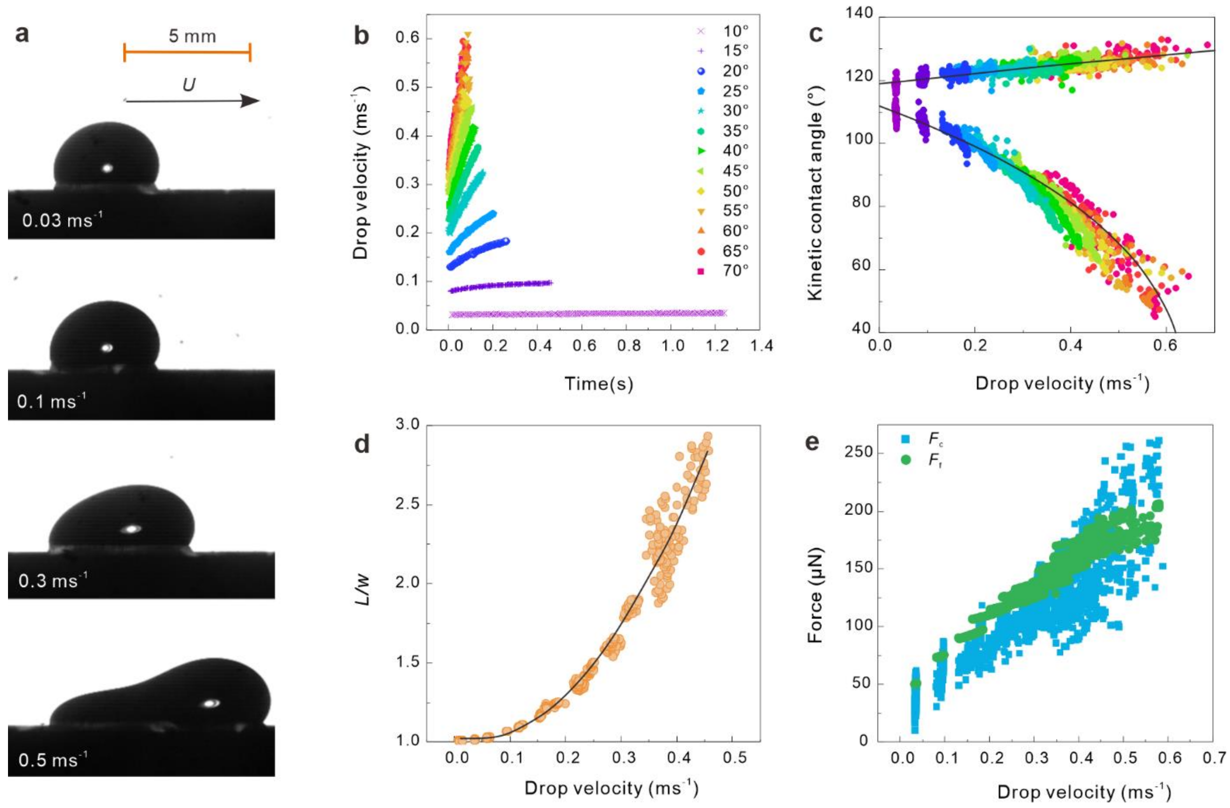

Supplementary Figure 10. 30% glycerol-water mixture drops on Teflon-gold surfaces. (a) Drop profile at different velocities; (b) velocity of drops at different tilt angles; (c) velocity-dependent kinetic contact angle (upper data point corresponds to the advancing side, below data point corresponds to the receding side). The black curves are fitted by $\theta_{\mathrm{a/r}} = \left(\theta^3_{\mathrm{a0/r0}} \pm 9\frac{U\eta}{\gamma}\ln\frac{l}{l_{\mathrm{m}}}\right)^{1/3}$. The fitting parameters ($\theta_{\mathrm{a0}}, \theta_{\mathrm{r0}}, \frac{l}{l_{\mathrm{m}}}$) are summarized in Supplementary Table 1; (d) velocity-dependent aspect ratio (length-to-width: L/w). The data was fitted with a polynom resulting in: $\frac{L}{w} = 10.33U^2 - 0.729U + 1.03$, U in ms^{-1}; (e) velocity-dependent friction force and capillary force.

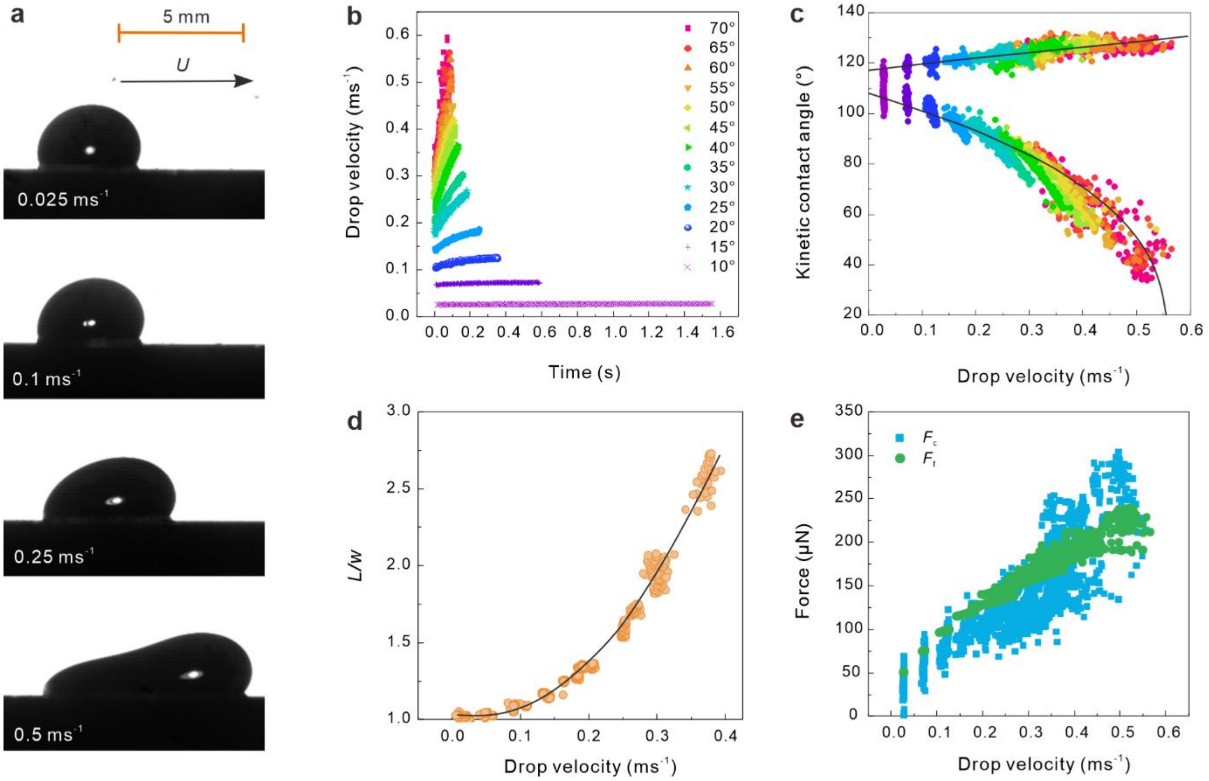

Supplementary Figure 11. 40% glycerol-water mixture drops on Teflon-gold surfaces. (a) Drop profile at different velocities; (b) velocity of drops at different tilt angles; (c) velocity-dependent kinetic contact angle (upper data point corresponds to the advancing side, below data point corresponds to the receding side). The black curves are fitted by $\theta_{\mathrm{a/r}} = \left(\theta_{\mathrm{a0/r0}}^3 \pm 9\frac{U\eta}{\gamma}\ln\frac{l}{l_{\mathrm{m}}}\right)^{1/3}$. The fitting parameters ($\theta_{\mathrm{a0}}, \theta_{\mathrm{r0}}, \frac{l}{l_{\mathrm{m}}}$) are summarized in Supplementary Table 1; (d) velocity-dependent aspect ratio (length-to-width: L/w). The data was fitted with a polynom resulting in: $\frac{L}{w} = 13.68U^2 - 1.087U + 1.043$, U in ms^{-1}; (e) velocity-dependent friction force and capillary force.

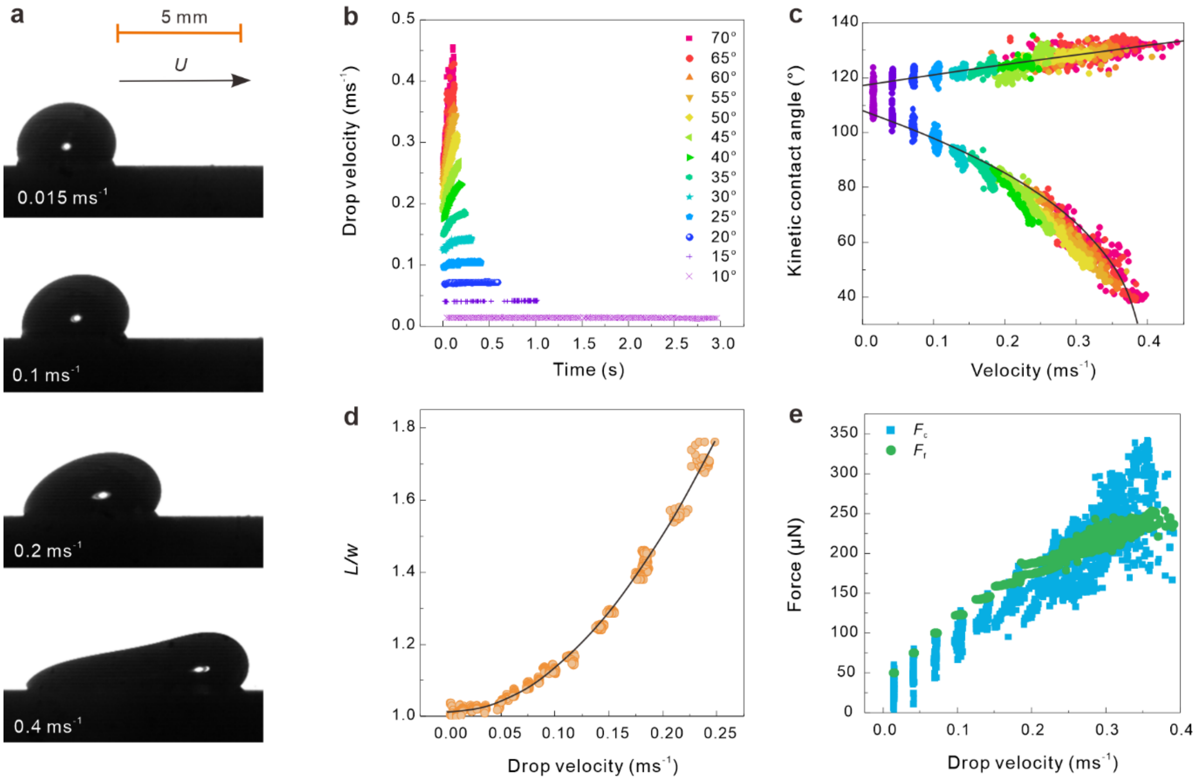

Supplementary Figure 12. 50% glycerol-water mixture drops on Teflon-gold surfaces. (a) Drop profile at different velocities; (b) velocity of drops at different tilt angles; (c) velocity-dependent kinetic contact angle (upper data point corresponds to the advancing side, below data point corresponds to the receding side). The black curves are fitted by $\theta_{\mathrm{a/r}} = \left(\theta^3_{\mathrm{a0/r0}} \pm 9\frac{U\eta}{\gamma}\ln\frac{l}{l_{\mathrm{m}}}\right)^{1/3}$. The fitting parameters $(\theta_{\mathrm{a0}}, \theta_{\mathrm{r0}}, \frac{l}{l_{\mathrm{m}}})$ are summarized in Supplementary Table 1; (d) velocity-dependent aspect ratio (length-to-width: L/w). The data was fitted with a polynom resulting in: $\frac{L}{w} = 12.12U^2 - 0.001U + 1.013$, U in ms^{-1}; (e) velocity-dependent friction force and capillary force.

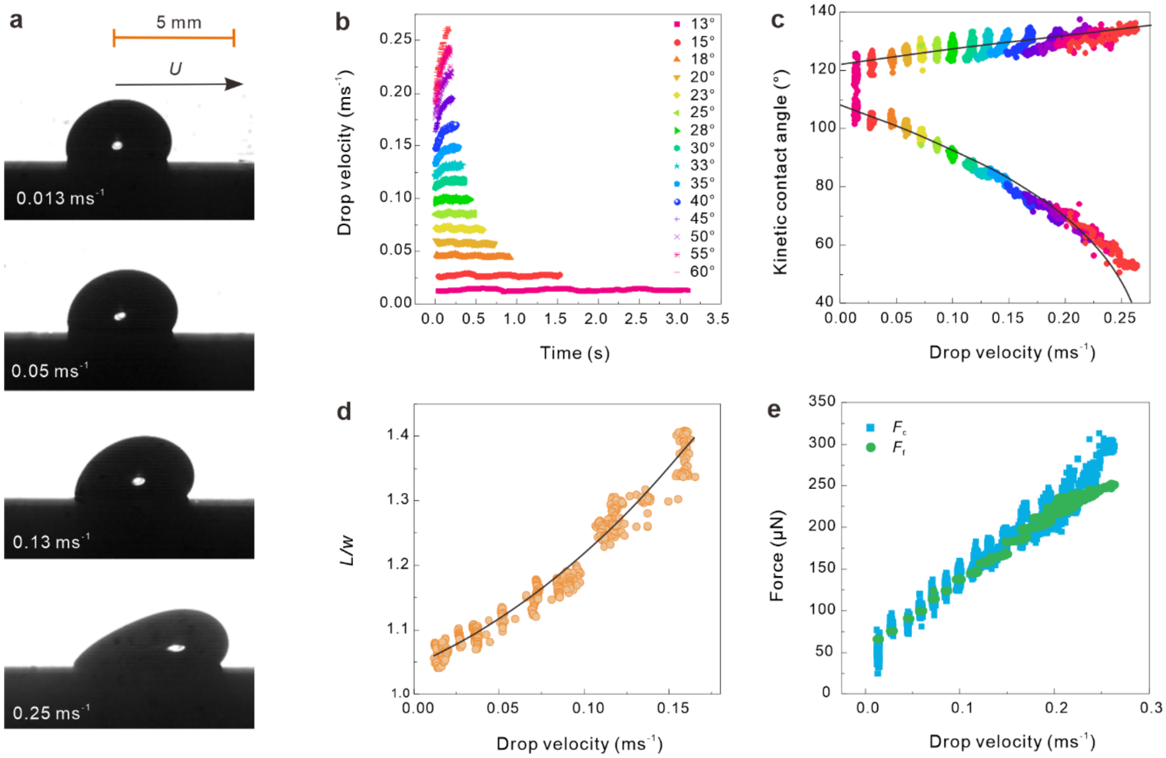

Supplementary Figure 13. 60% glycerol-water mixture drops on Teflon-gold surfaces. (a) Drop profile at different velocities; (b) velocity of drops at different tilt angles; (c) velocity-dependent kinetic contact angle (upper data point corresponds to the advancing side, below data point corresponds to the receding side). The black curves are fitted by $\theta_{\mathrm{a/r}} = \left(\theta_{\mathrm{a0/r0}}^3 \pm 9\frac{U\eta}{\gamma}\ln\frac{l}{l_{\mathrm{m}}}\right)^{1/3}$. The fitting parameters ($\theta_{\mathrm{a0}}, \theta_{\mathrm{r0}}, \frac{l}{l_{\mathrm{m}}}$) are summarized in Supplementary Table 1; (d) velocity-dependent aspect ratio (length-to-width: L/w). The data was fitted with a polynom resulting in $\frac{L}{w} = 6.406U^2 + 1.076U + 1.047$, U in ms^{-1}; (e) velocity-dependent friction force and capillary force.

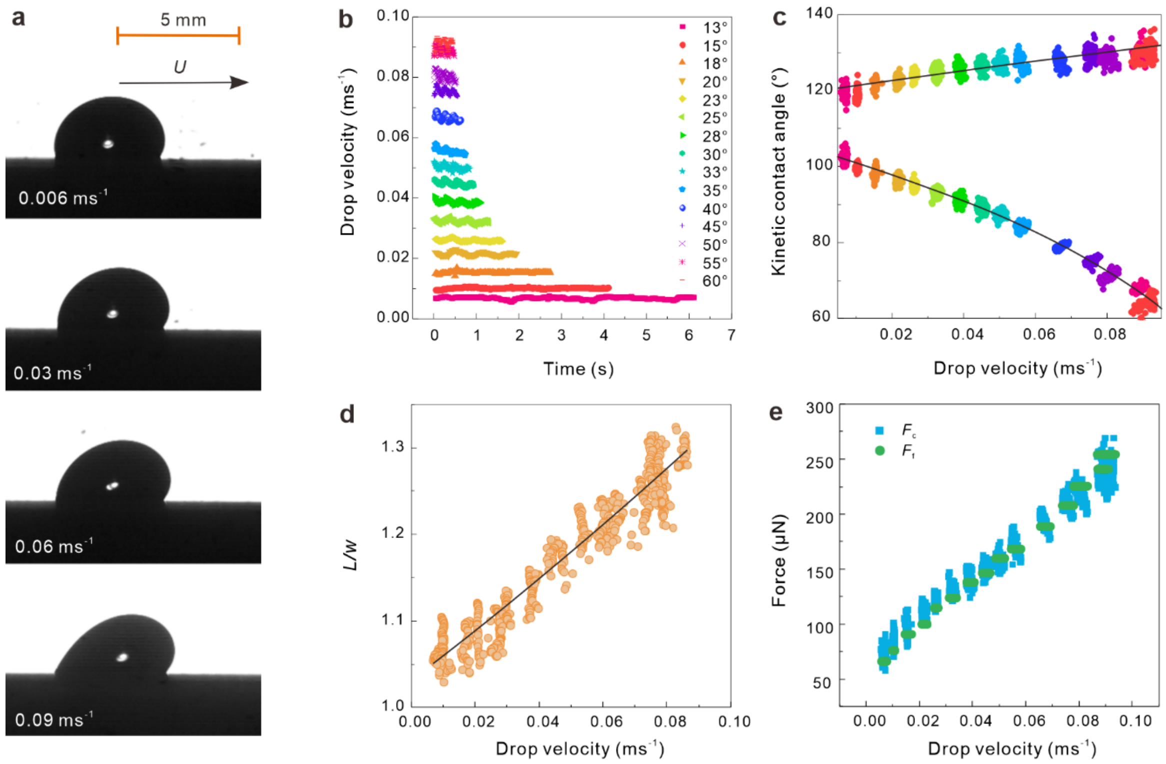

Supplementary Figure 14. 70% glycerol-water mixture drops on Teflon-gold surfaces. (a) Drop profile at different velocities; (b) velocity of drops at different tilt angles; (c) velocity-dependent kinetic contact angle (upper data point corresponds to the advancing side, below data point corresponds to the receding side). The black curves are fitted by $\theta_{\mathrm{a/r}} = \left(\theta_{\mathrm{a0/r0}}^{3} \pm 9\frac{U\eta}{\gamma}\ln\frac{l}{l_{\mathrm{m}}}\right)^{1/3}$. The fitting parameters ($\theta_{\mathrm{a0}}, \theta_{\mathrm{r0}}, \frac{l}{l_{\mathrm{m}}}$) are summarized in Supplementary Table 1; (d) velocity-dependent aspect ratio (length-to-width: L/w). The data was fitted with a polynom resulting in $\frac{L}{w} = 3.17U^2 + 2.80U + 1.03$, U in ms^{-1}; (e) velocity-dependent friction force and capillary force.

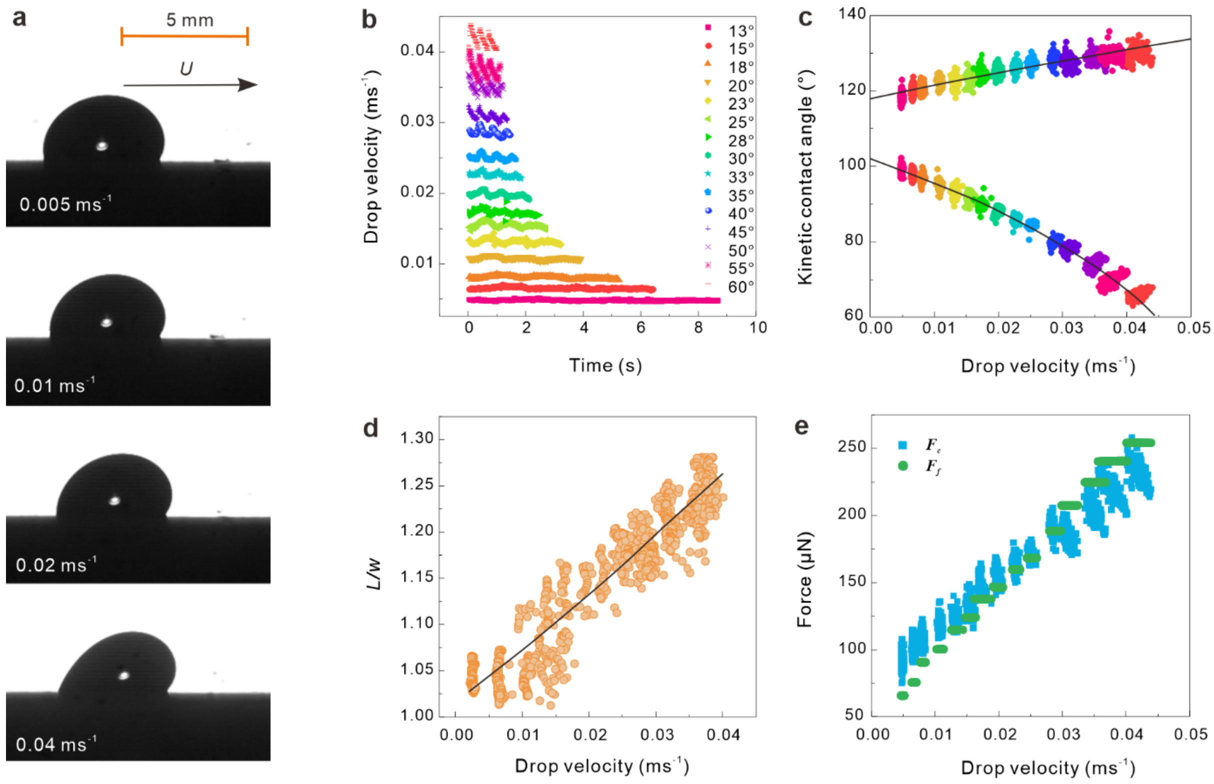

Supplementary Figure 15. 80% glycerol-water mixture drops on Teflon-gold surfaces. (a) Drop profile at different velocities; (b) velocity of drops at different tilt angles; (c) velocity-dependent kinetic contact angle (upper data point corresponds to the advancing side, below data point corresponds to the receding side). The black curves are fitted by $\theta_{\mathrm{a/r}} = \left(\theta_{\mathrm{a0/r0}}^3 \pm 9\frac{U\eta}{\gamma}\ln\frac{l}{l_{\mathrm{m}}}\right)^{1/3}$. The fitting parameters $(\theta_{\mathrm{a0}}, \theta_{\mathrm{r0}}, \frac{l}{l_{\mathrm{m}}})$ are summarized in Supplementary Table 1; (d) velocity-dependent aspect ratio (length-to-width: L/w). The data was fitted with a polynom resulting in $\frac{L}{w} = 17.85U^2 + 5.46U + 1.02$, U in ms^{-1}; (e) velocity-dependent friction force and capillary force.

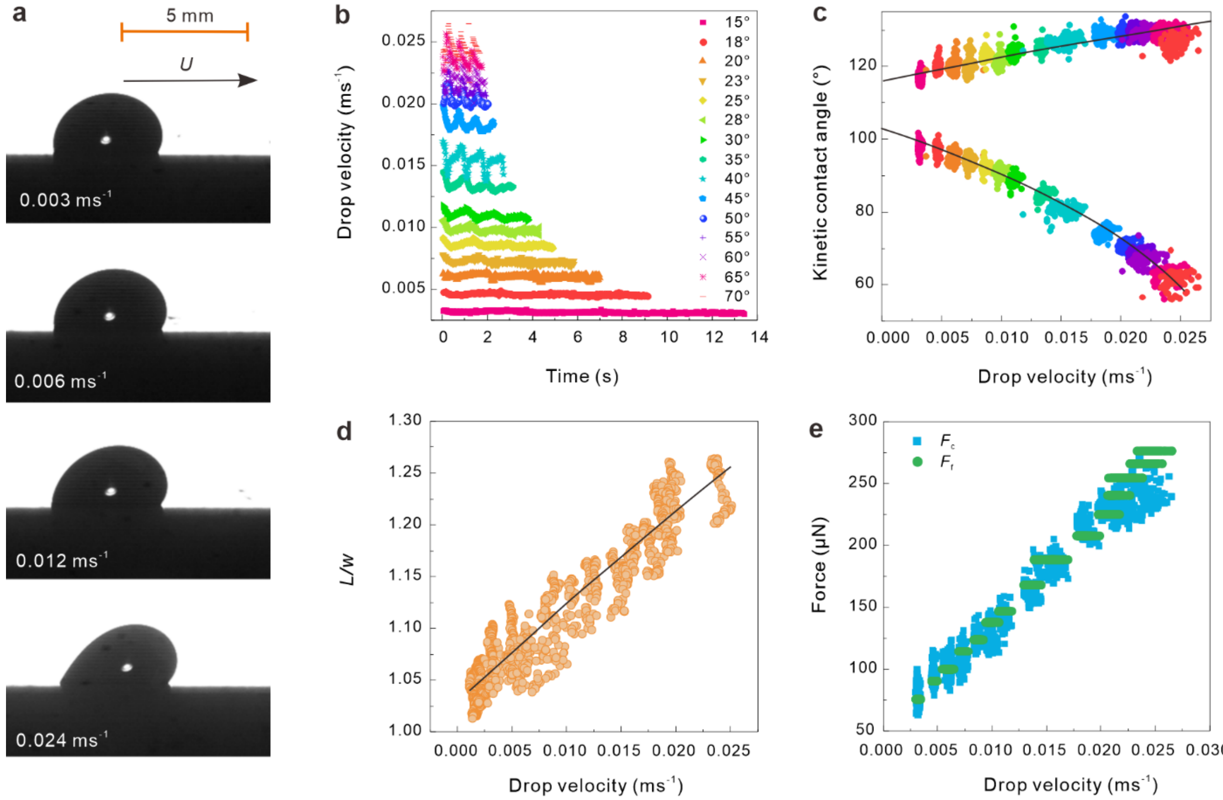

Supplementary Figure 16. 85% glycerol-water mixture drops on Teflon-gold surfaces. (a) Drop profile at different velocities; (b) velocity of drops at different tilt angles; (c) velocity-dependent kinetic contact angle (upper data point corresponds to the advancing side, below data point corresponds to the receding side). The black curves are fitted by $\theta_{\mathrm{a/r}} = \left(\theta_{\mathrm{a0/r0}}^{3} \pm 9\frac{U\eta}{\gamma}\ln\frac{l}{l_{\mathrm{m}}}\right)^{1/3}$. The fitting parameters $(\theta_{\mathrm{a0}}, \theta_{\mathrm{r0}}, \frac{l}{l_{\mathrm{m}}})$ are summarized in Supplementary Table 1; (d) velocity-dependent aspect ratio (length-to-width: L/w). The data was fitted with a polynom resulting in $\frac{L}{w} = -29.44U^2 + 9.83U + 1.03$, U in ms^{-1}; (e) velocity-dependent friction force and capillary force.

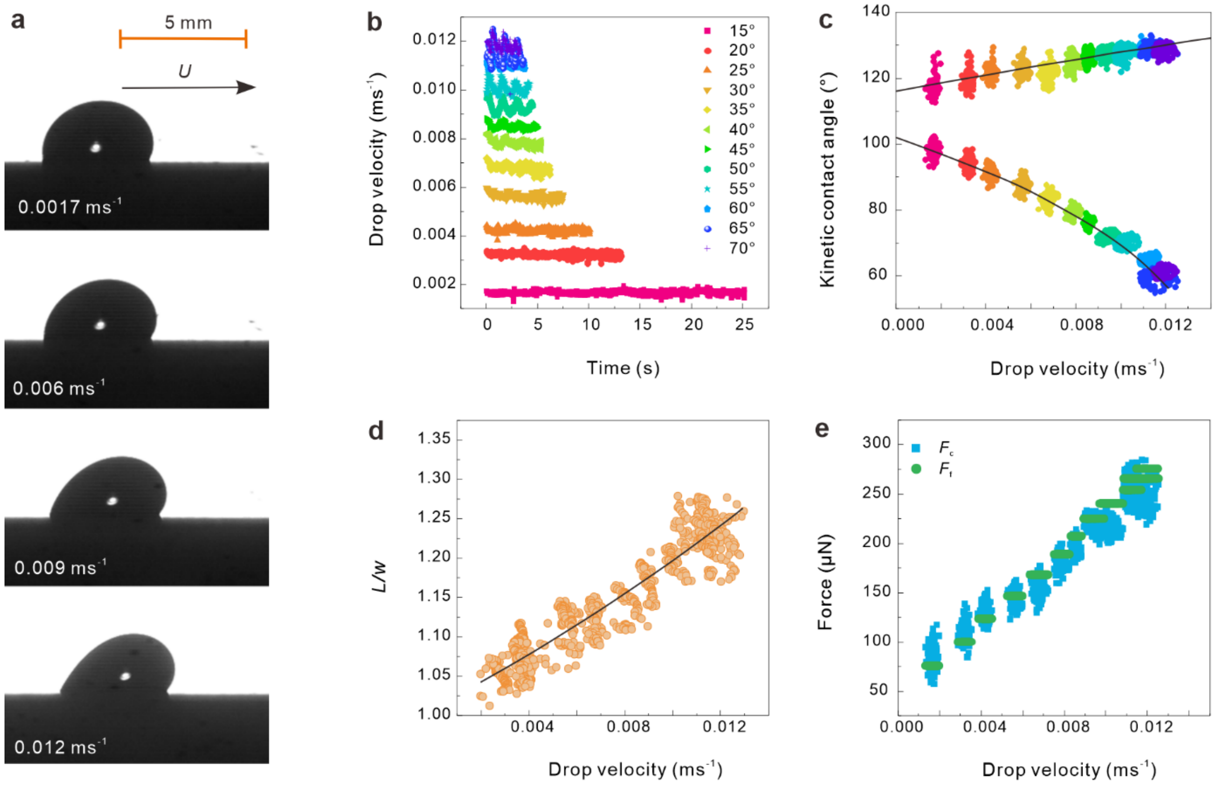

Supplementary Figure 17. 90% glycerol-water mixture drops on Teflon-gold surfaces. (a) Drop profile at different velocities; (b) velocity of drops at different tilt angles; (c) velocity-dependent kinetic contact angle (upper data point corresponds to the advancing side, below data point corresponds to the receding side). The black curves are fitted by $\theta_{\mathrm{a/r}} = \left(\theta^3_{\mathrm{a0/r0}} \pm 9\frac{U\eta}{\gamma}\ln\frac{l}{l_{\mathrm{m}}}\right)^{1/3}$. The fitting parameters $(\theta_{\mathrm{a0}}, \theta_{\mathrm{r0}}, \frac{l}{l_{\mathrm{m}}})$ are summarized in Supplementary Table 1; (d) velocity-dependent aspect ratio (length-to-width: L/w). The data was fitted with a polynom resulting in $\frac{L}{w} = 315.2U^2 + 15.38U + 1.01$, U in ms^{-1}; (e) velocity-dependent friction force and capillary force.

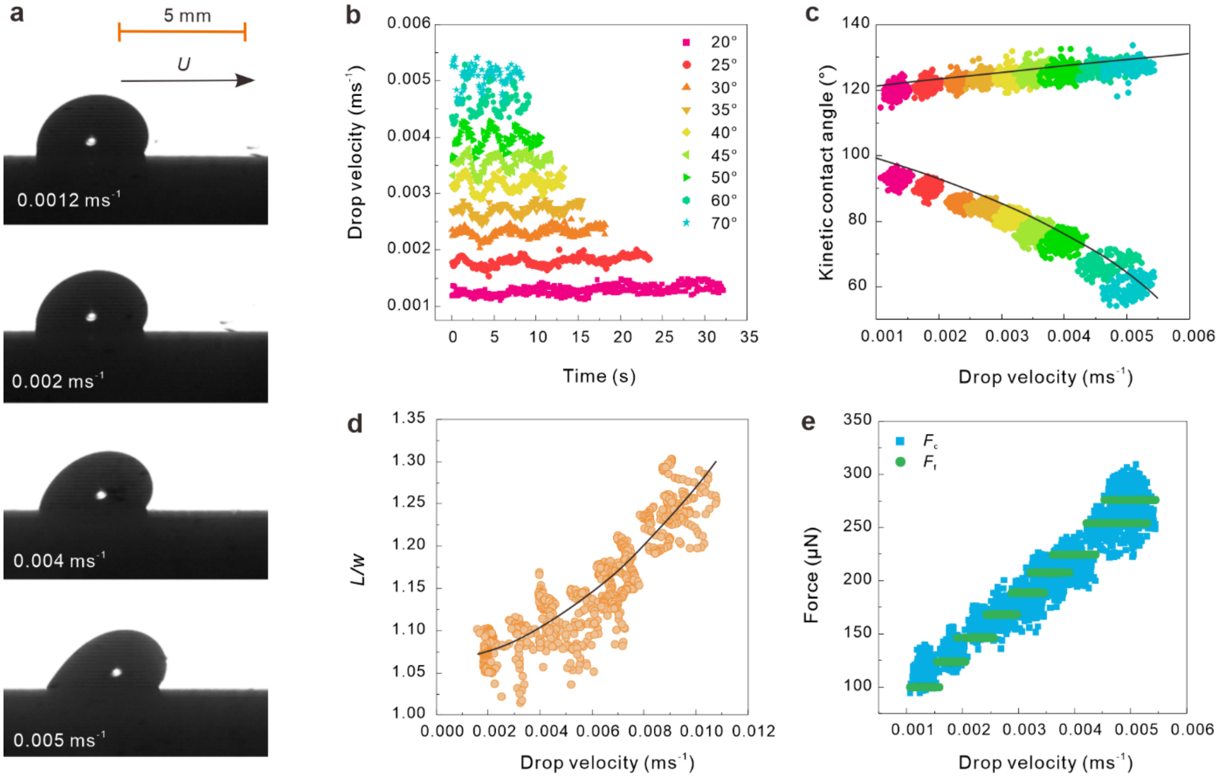

Supplementary Figure 18. 95% glycerol-water mixture drops on Teflon-gold surfaces. (a) Drop profile at different velocities; (b) velocity of drops at different tilt angles; (c) velocity-dependent kinetic contact angle (upper data point corresponds to the advancing side, below data point corresponds to the receding side). The black curves are fitted by $\theta_{\mathrm{a/r}} = \left(\theta_{\mathrm{a0/r0}}^3 \pm 9\frac{U\eta}{\gamma}\ln\frac{l}{l_{\mathrm{m}}}\right)^{1/3}$. The fitting parameters $(\theta_{\mathrm{a0}}, \theta_{\mathrm{r0}}, \frac{l}{l_{\mathrm{m}}})$ are summarized in Supplementary Table 1; (d) velocity-dependent aspect ratio (length-to-width: L/w). The data was fitted with a polynom resulting in $\frac{L}{w} = 1696U^2 + 3.87U + 1.06$, U in ms^{-1}; (e) velocity-dependent friction force and capillary force.

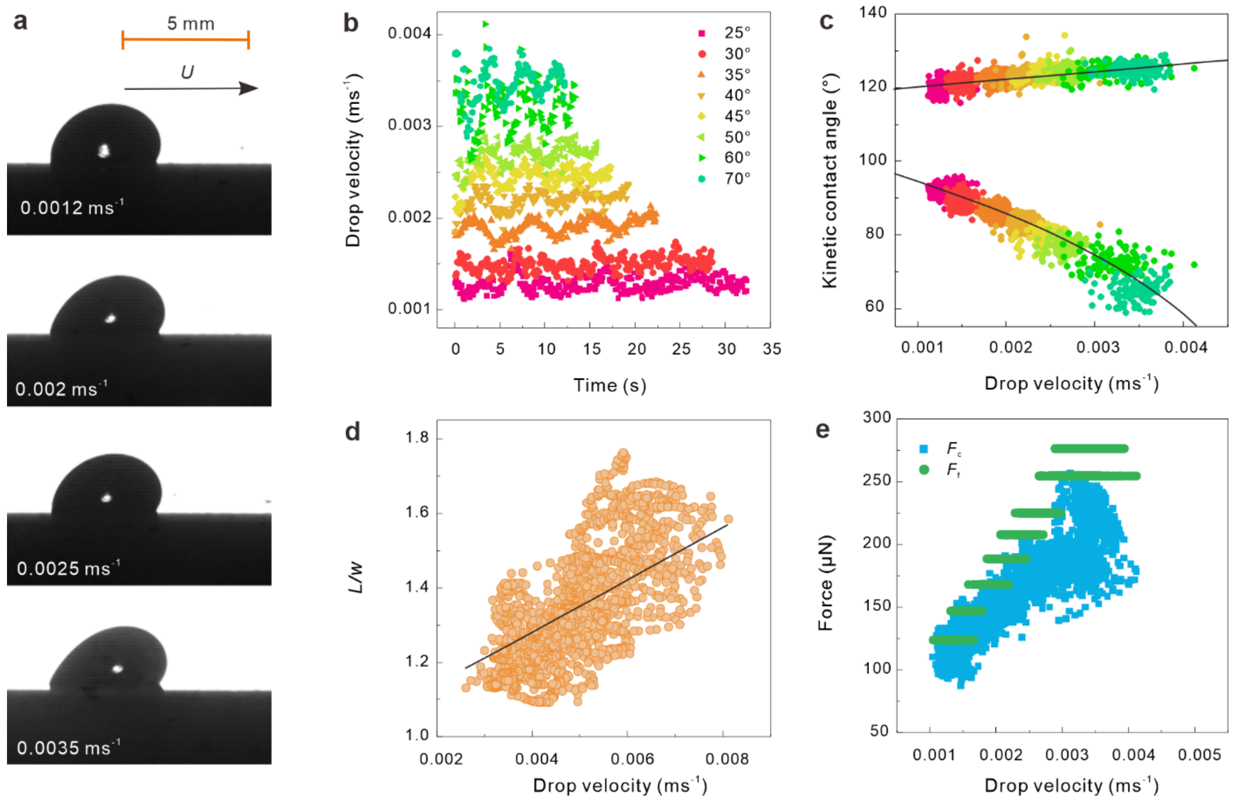

Supplementary Figure 19. 99% glycerol-water mixture drops on Teflon-gold surfaces. (a) Drop profile at different velocities; (b) velocity of drops at different tilt angles; (c) velocity-dependent kinetic contact angle (upper data point corresponds to the advancing side, below data point corresponds to the receding side). The black curves are fitted by $\theta_{\mathrm{a/r}} = \left(\theta_{\mathrm{a0/r0}}^3 \pm 9\frac{U\eta}{\gamma}\ln\frac{l}{l_{\mathrm{m}}}\right)^{1/3}$. The fitting parameters $(\theta_{\mathrm{a0}}, \theta_{\mathrm{r0}}, \frac{l}{l_{\mathrm{m}}})$ are summarized in Supplementary Table 1; (d) velocity-dependent aspect ratio (length-to-width: L/w). The data was fitted with a polynom resulting in $\frac{L}{w} = 70.25U + 1$, U in ms^{-1}; (e) velocity-dependent friction force and capillary force.

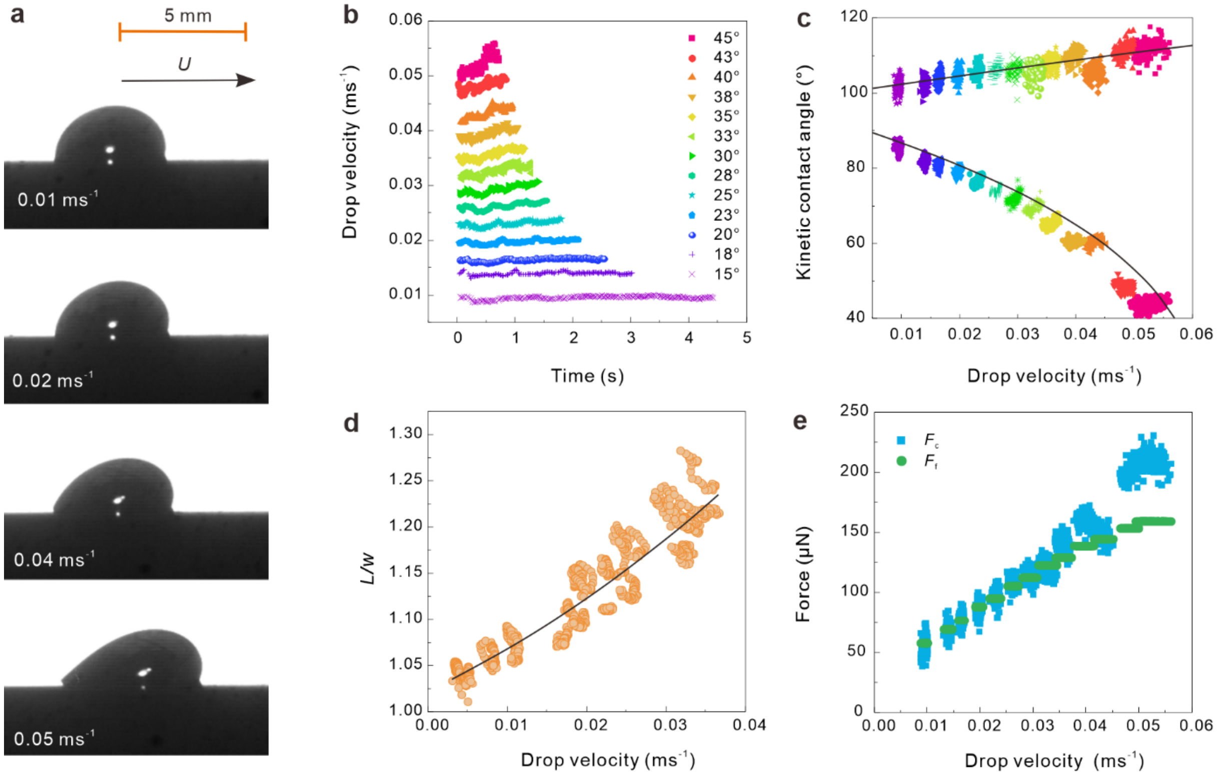

Supplementary Figure 20. Ethylene glycol drops on Teflon-gold surfaces. (a) Drop profile at different velocities; (b) velocity of drops at different tilt angles; (c) velocity-dependent kinetic contact angle (upper data point corresponds to the advancing side, below data point corresponds to the receding side). The black curves are fitted by $\theta_{\mathrm{a/r}} = \left(\theta_{\mathrm{a0/r0}}^{3} \pm 9\frac{U\eta}{\gamma}\ln\frac{l}{l_{\mathrm{m}}}\right)^{1/3}$. The fitting parameters ($\theta_{\mathrm{a0}}, \theta_{\mathrm{r0}}, \frac{l}{l_{\mathrm{m}}}$) are summarized in Supplementary Table 1; (d) velocity-dependent aspect ratio (length-to-width: L/w). The data was fitted with a polynom resulting in $\frac{L}{w} = 49.04U^2 + 3.99U + 1.02$, U in ms^{-1}; (e) velocity-dependent friction force and capillary force.

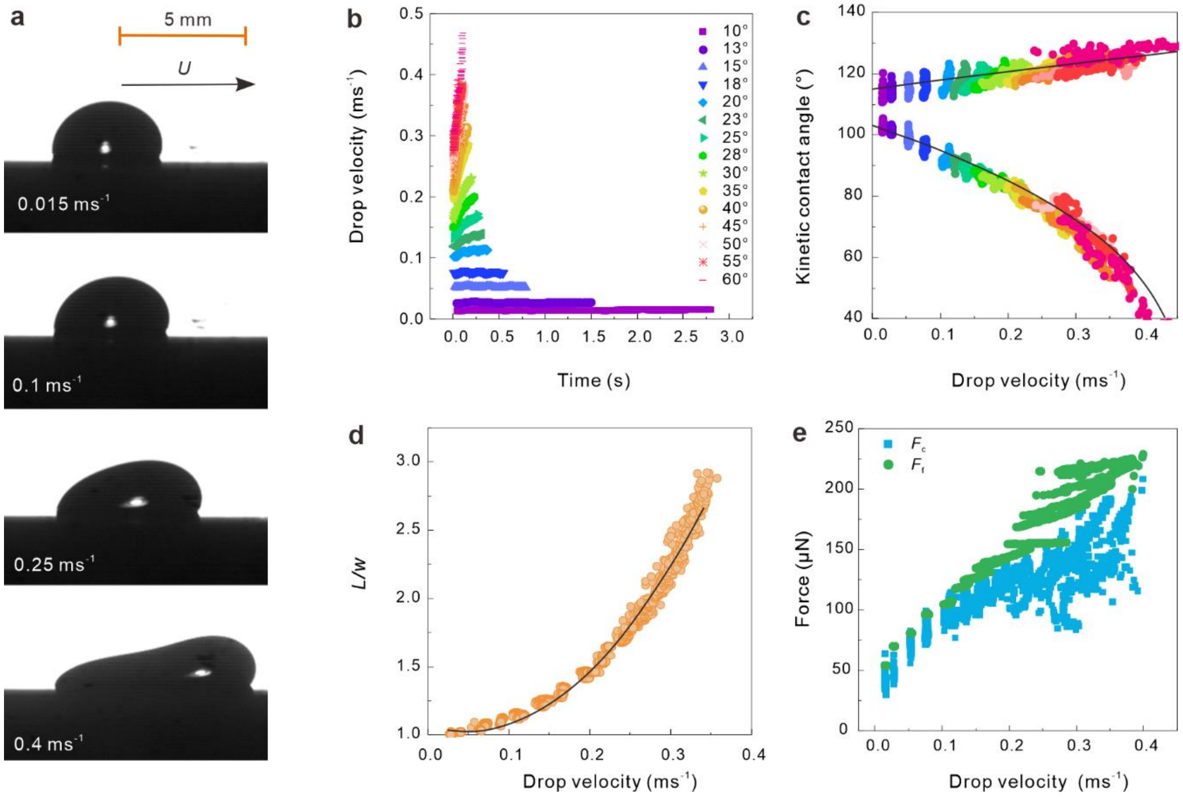

Supplementary Figure 21. Formamide drops on Teflon-gold surfaces. (a) Drop profile at different velocities; (b) velocity of drops at different tilt angles; (c) velocity-dependent kinetic contact angle (upper data point corresponds to the advancing side, below data point corresponds to the receding side). The black curves are fitted by $\theta_{\mathrm{a/r}} = \left(\theta_{\mathrm{a0/r0}}^{3} \pm 9\frac{U\eta}{\gamma}\ln\frac{l}{l_{\mathrm{m}}}\right)^{1/3}$. The fitting parameters ($\theta_{\mathrm{a0}}, \theta_{\mathrm{r0}}, \frac{l}{l_{\mathrm{m}}}$) are summarized in Supplementary Table 1; (d) velocity-dependent aspect ratio (length-to-width: L/w). The data was fitted with a polynom resulting in $\frac{L}{w} = 19.46U^2 - 2.00U + 1.08$, U in ms^{-1}; (e) velocity-dependent friction force and capillary force.

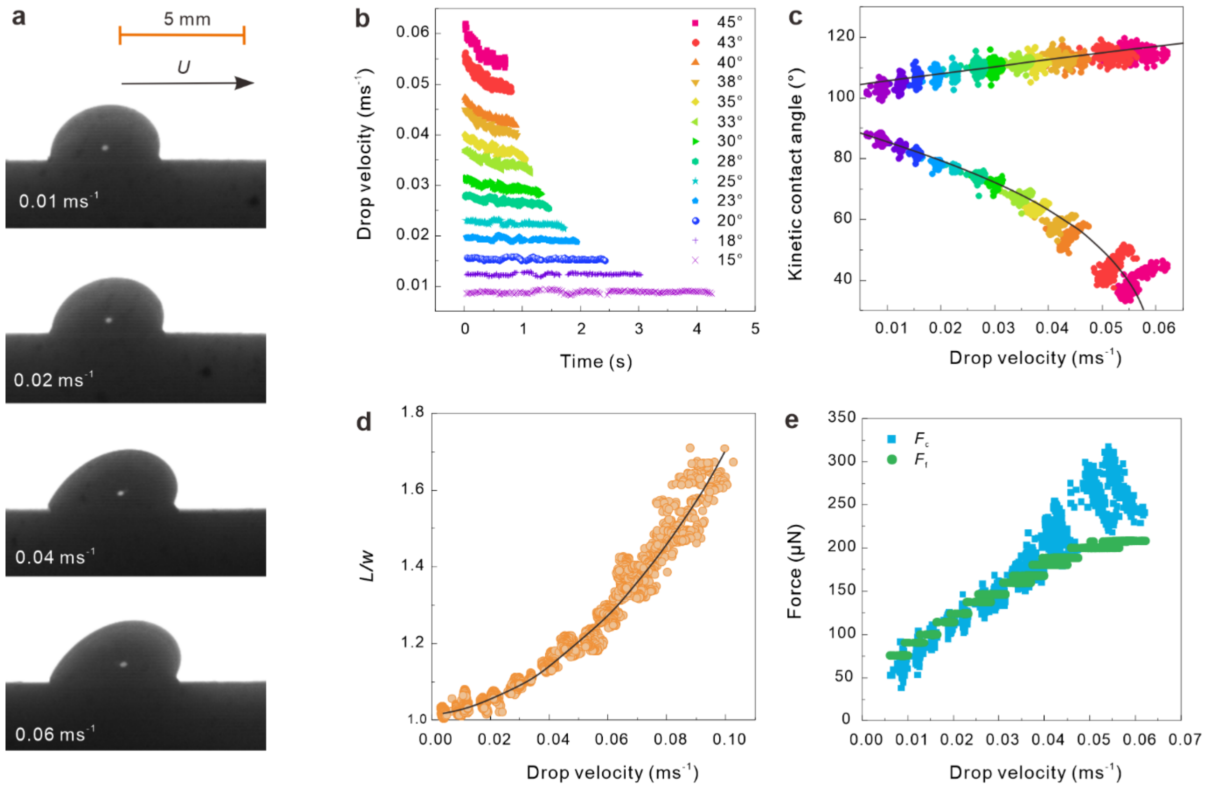

Supplementary Figure 22. Ionic liquid drops on Teflon-gold surfaces. (a) Drop profile at different velocities; (b) velocity of drops at different tilt angles; (c) velocity-dependent kinetic contact angle (upper data point corresponds to the advancing side, below data point corresponds to the receding side). The black curves are fitted by $\theta_{\mathrm{a/r}} = \left(\theta_{\mathrm{a0/r0}}^3 \pm 9\frac{U\eta}{\gamma}\ln\frac{l}{l_{\mathrm{m}}}\right)^{1/3}$. The fitting parameters $(\theta_{\mathrm{a0}}, \theta_{\mathrm{r0}}, \frac{l}{l_{\mathrm{m}}})$ are summarized in Supplementary Table 1; (d) velocity-dependent aspect ratio (length-to-width: L/w). The data was fitted with a polynom resulting in $\frac{L}{w} = 62.06U^2 + 0.599U + 1.02$, U in ms-1; (e) velocity-dependent friction force and capillary force.

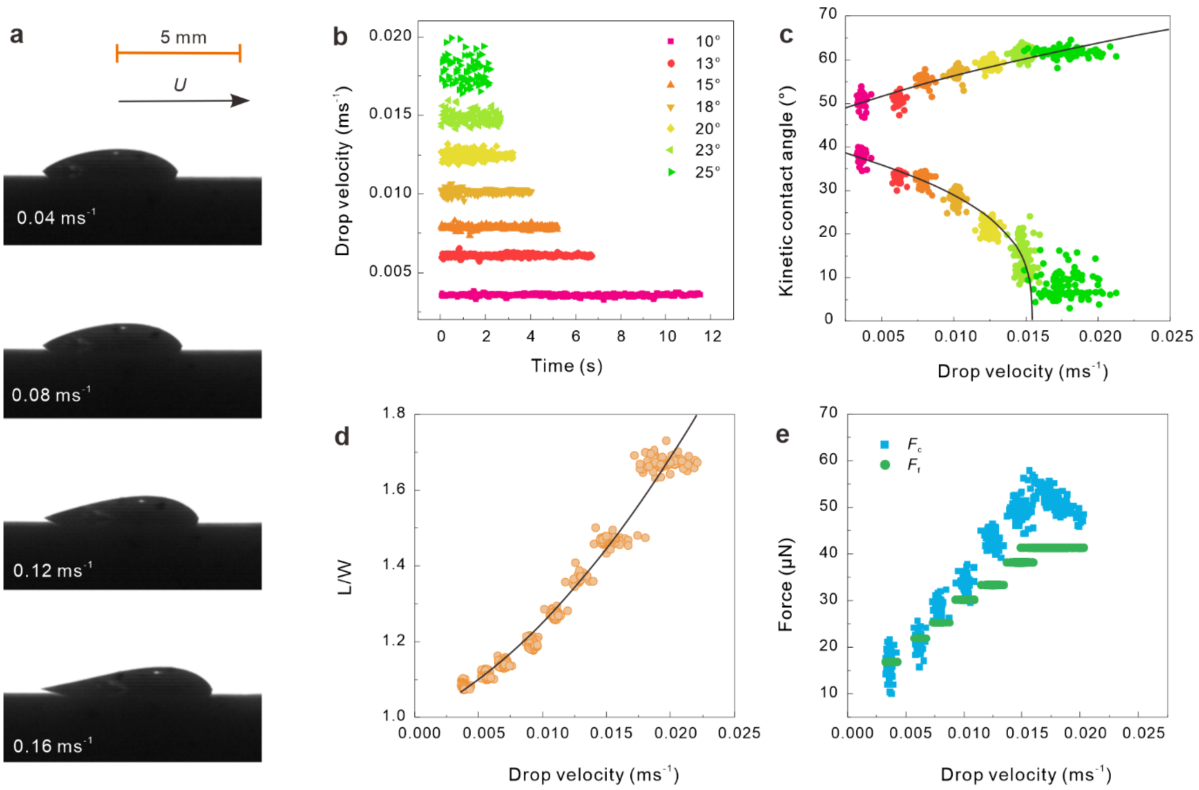

Supplementary Figure 23. 5 cSt silicone oil drops on Teflon-gold surfaces. (a) Drop profile at different velocities; (b) velocity of drops at different tilt angles; (c) velocity-dependent kinetic contact angle (upper data point corresponds to the advancing side, below data point corresponds to the receding side). The black curves are fitted by $\theta_{\mathrm{a/r}} = \left(\theta_{\mathrm{a0/r0}}^3 \pm 9\frac{U\eta}{\gamma}\ln\frac{l}{l_{\mathrm{m}}}\right)^{1/3}$. The fitting parameters ($\theta_{\mathrm{a0}}, \theta_{\mathrm{r0}}, \frac{l}{l_{\mathrm{m}}}$) are summarized in Supplementary Table 1; (d) velocity-dependent aspect ratio (length-to-width: L/w). The data was fitted with a polynom resulting in $\frac{L}{w} = 921.2U^2 + 16.02U + 0.997$, U in ms^{-1}; (e) velocity-dependent friction force and capillary force.

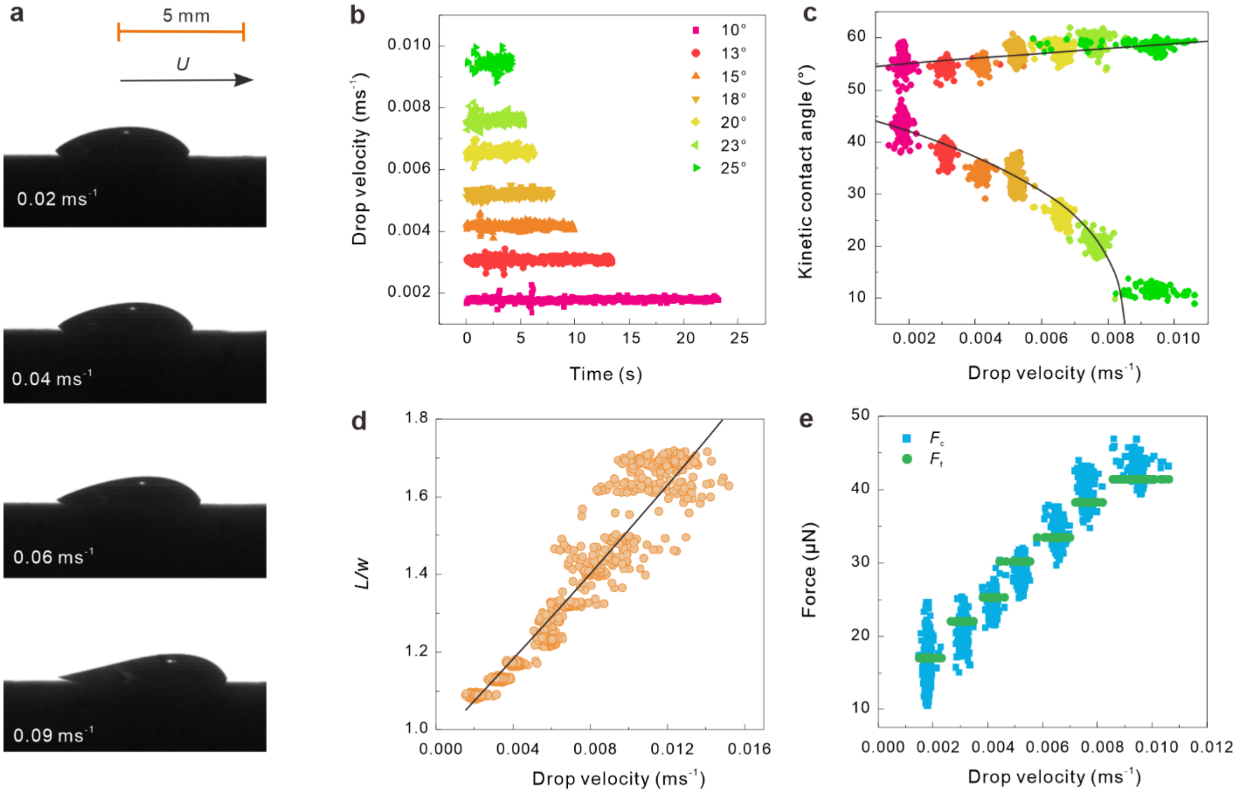

Supplementary Figure 24. 10 cSt silicone oil drops on Teflon-gold surfaces. (a) Drop profile at different velocities; (b) velocity of drops at different tilt angles; (c) velocity-dependent kinetic contact angle (upper data point corresponds to the advancing side, below data point corresponds to the receding side). The black curves are fitted by $\theta_{\mathrm{a/r}} = \left(\theta^3_{\mathrm{a0/r0}} \pm 9\frac{U\eta}{\gamma}\ln\frac{l}{l_\mathrm{m}}\right)^{1/3}$. The fitting parameters ($\theta_{\mathrm{a0}}, \theta_{\mathrm{r0}}, \frac{l}{l_\mathrm{m}}$) are summarized in Supplementary Table 1; (d) velocity-dependent aspect ratio (length-to-width: L/w). The data was fitted with a polynom resulting in $\frac{L}{w} = 256.4U^2 + 51.7U + 0.971$, U in ms-1; (e) velocity-dependent friction force and capillary force.

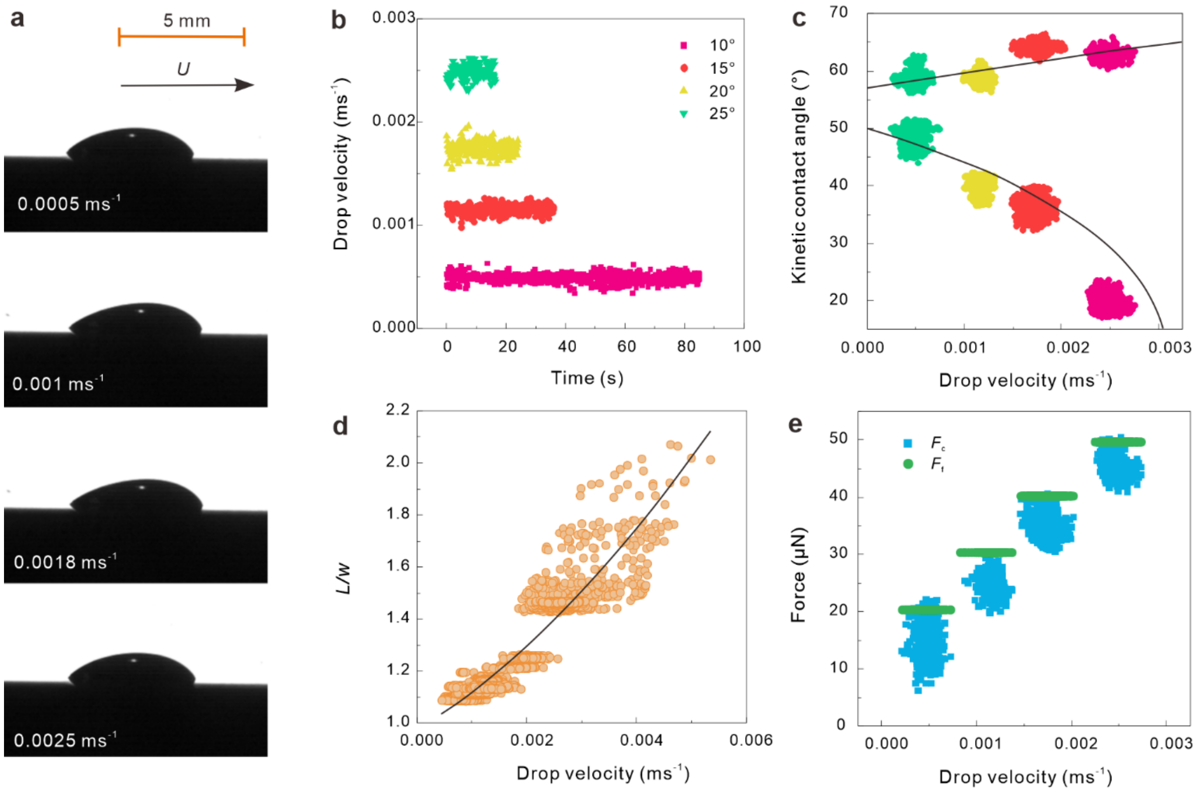

Supplementary Figure 25. 50 cSt silicone oil drops on Teflon-gold surfaces. (a) Drop profile at different velocities; (b) velocity of drops at different tilt angles; (c) velocity-dependent kinetic contact angle (upper data point corresponds to the advancing side, below data point corresponds to the receding side). The black curves are fitted by $\theta_{\mathrm{a/r}} = \left(\theta^3_{\mathrm{a0/r0}} \pm 9\frac{U\eta}{\gamma}\ln\frac{l}{l_{\mathrm{m}}}\right)^{1/3}$. The fitting parameters ($\theta_{\mathrm{a0}}, \theta_{\mathrm{r0}}, \frac{l}{l_{\mathrm{m}}}$) are summarized in Supplementary Table 1; (d) velocity-dependent aspect ratio (length-to-width: L/w). The data was fitted with a polynom resulting in $\frac{L}{w} = 16420U^2 + 127.3U + 0.974$, U in ms^{-1}; (e) velocity-dependent friction force and capillary force.

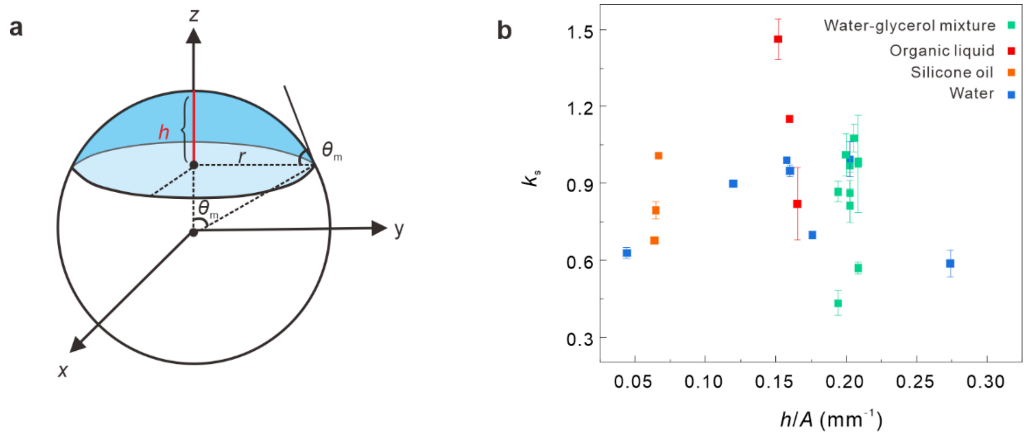

Supplementary Figure 26. The correlation between static k-factor (k_s) and h/A (h is the height of the drop while A is the liquid-solid contact area). (a) Schematics of a spherical cap. By assuming the static drop is a spherical cap with contact radius r, we estimated the drop height by $h = r(1-\cos\theta_m)/\sin\theta_m$ ($\theta_m = \frac{\theta_{as}+\theta_{rs}}{2}$ is the mean contact angle) and the contact area by $A = \pi r^2$. Knowing the drop volume V, the contact radius was calculated with $r=\sqrt[3]{\frac{3V\sin^3\theta_m}{\pi(1-\cos\theta_m)^2(2+\cos\theta_m)}}$. Finally, we calculated h/A based on the mean contact angle and drop volume. (b) The plot of k_s versus h/A. Error bars indicate Gaussian error propagation of the standard deviation of $\theta_{as/rs}$ and F_0/w.

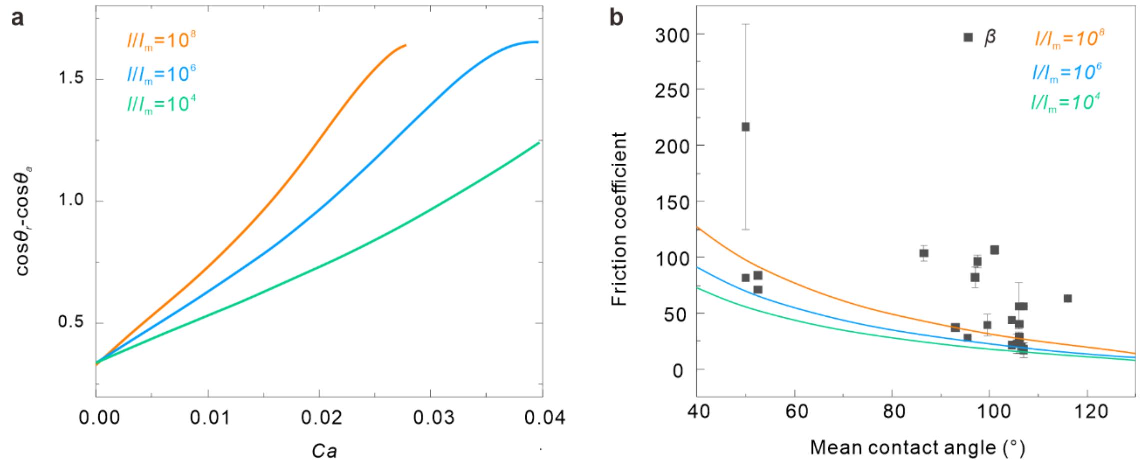

Supplementary Figure 27. Hydrodynamic hypothesis. (a) Capillary forces scaled by the width of the contact area, the surface tension of the liquid, and the geometry factor ($\frac{F_c}{k\gamma w} = \cos\theta_r - \cos\theta_a$) versus capillary number (Ca). By assuming $\theta_{a/r} = \left(\theta_{a0/r0}^3 \pm 9\frac{U\eta}{\gamma}\ln\frac{l}{l_m}\right)^{1/3}$, we calculated $\cos\theta_r - \cos\theta_a$ with

different ratios of the macroscopic-to-microscopic length scale, l/l_{m}. (b) The correlation between friction coefficient and contact angle. The solid curves present the theoretical correlation between the mean contact angle (θ_{m}) and friction coefficient based on $\beta' = 6k \ln \frac{l}{l_{\mathrm{m}}} \cdot \left(\frac{\sin\theta_{\mathrm{m}}}{\theta_{\mathrm{m}}^2}\right)$ when inserting $l/l_{\mathrm{m}} = 10^4$ (green), 10^6 (blue), and 10^8 (orange). Error bars indicate the standard deviation of β from two measurements.

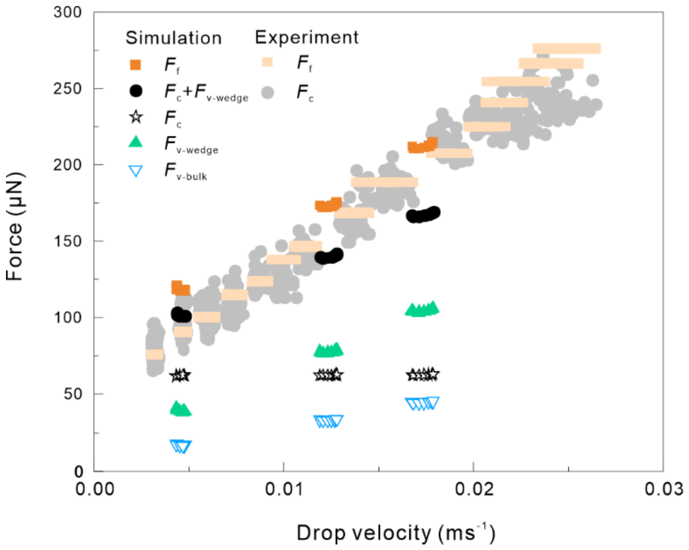

Supplementary Figure 28. Comparison between experimental force and simulated force for sliding drops with 85% glycerol-water mixture on Teflon-gold surfaces. F_{f}, F_{c}, $F_{\mathrm{v-wedge}}$, and $F_{\mathrm{v-bulk}}$ are friction force, capillary force, wedge viscous force, and bulk viscous force.

Supplementary Table 1. Fitting parameters ($\theta_{\mathrm{a0}}, \theta_{\mathrm{ro}}$, and $\frac{l}{l_{\mathrm{m}}}$) when fitting the velocity-dependent kinetic contact angles by $\theta_{\mathrm{a/r}} = \left(\theta_{\mathrm{a0/r0}}^{3} \pm 9\frac{U\eta}{\gamma}\ln\frac{l}{l_{\mathrm{m}}}\right)^{1/3}$ (Cox-Voinov theory) in Fig. 4g and Supplementary Figure 4-25c. Note: (1) θ_{a0} and θ_{ro} are microscopically advancing and receding contact angles in the Cox-Voinov theory, different from static advancing and receding contact angles ($\theta_{\mathrm{as/rs}}$) in Table 1. (2) l/l_{m} is the ratio of macroscopic length scale (l) to microscopic length (l_{m}) scale. We fitted the velocity-dependent advancing and receding contact angles separately, therefore, $\frac{l}{l_{\mathrm{m}}}$ for the advancing and receding sides is different.

System number	Liquid-Surface	γ mN/m	η $\mathrm{mPa \cdot s}$	θ_{a0} (1) °	θ_{r0} (1) °	$(l/l_{\mathrm{m}})_{\mathrm{a}}$ (2) (Advancing)	$(l/l_{\mathrm{m}})_{\mathrm{r}}$ (2) (Receding)
1	Water-Si wafer	72	0.92	44	26	1.1×10^{24}	1.5×10^{7}
2	Water-ITO glass	72	0.92	118	91	2.4×10^{3}	5.2×10^{23}
3	Water-PFOTS	72	0.92	106	90	3.3×10^{14}	1.9×10^{18}
4	Water-PDMS	72	0.92	110	91	9.0×10^{12}	1.2×10^{26}
5	Water-PS	72	0.92	95	82	2.5×10^{8}	5.2×10^{17}
6	Water-Thiols	72	0.92	132	107	4.3×10^{10}	2.9×10^{26}
7	Water-Teflon	72	0.92	125	119	2.8×10^{6}	1.5×10^{25}
8	30% Glycerol-Teflon	69	2.5	119	112	8.0×10^{4}	2.1×10^{15}
9	40% Glycerol-Teflon	69	3.8	117	108	7.8×10^{4}	3.2×10^{10}
10	50% Glycerol-Teflon	68	6.9	117	108	2.2×10^{4}	1.2×10^{8}
11	60% Glycerol-Teflon	67	13.6	122	108	1.2×10^{3}	6.7×10^{5}
12	70% Glycerol-Teflon	66	27.1	120	104	5.5×10^{3}	6.0×10^{5}
13	80% Glycerol-Teflon	66	75.9	118	102	2.2×10^{3}	1.7×10^{4}
14	85% Glycerol-Teflon	65	93	116	103	8.9×10^{4}	2.1×10^{6}
15	90% Glycerol-Teflon	65	192	116	102	4.6×10^{4}	2.2×10^{6}
16	95% Glycerol-Teflon	65	265	119	105	9.3×10^{5}	1.6×10^{11}
17	99% Glycerol-Teflon	64	943	118	102	4.6×10^{1}	5.6×10^{3}
18	Ethylene glycol-Teflon	48	16	100	90	3.3×10^{5}	4.9×10^{9}

19	Formamide-Teflon	58	4.6	115	103	7.5×10^{3}	6.1×10^{7}
20	Ionic liquid-Teflon	51	22	103	91	1.1×10^{5}	3.1×10^{7}
21	5 cSt silicone oil-Teflon	21	5	46	41	6.3×10^{8}	6.3×10^{4}
22	10 cSt silicone oil-Teflon	21	10	54	46	3.4×10^{2}	1.5×10^{6}
23	50 cSt silicone oil-Teflon	21	50	57	50	9.4×10^{2}	2.0×10^{4}
